# Hydroxychloroquine is neutral on incidental cataracts in patients with rheumatoid arthritis

**DOI:** 10.1038/s41598-023-32297-x

**Published:** 2023-04-05

**Authors:** Zhibo Zhang, Xin Ma, Yu-Hsun Wang, Xiaofei Shi, James Cheng-Chung Wei

**Affiliations:** 1 Department of Rheumatology and Immunology, The First Affiliated Hospital, College of Clinical Medicine, Henan University of Science and Technology, No. 24 Jinghua Road, Luoyang, China; 2 Department of Medical Research, Chung Shan Medical University Hospital, No. 110, Sec.1, Jianguo N. Rd., Taichung City, 40201 Taiwan; 3 Department of Allergy, Immunology and Rheumatology, Chung Shan Medical University Hospital, No. 110, Sec. 1, Jianguo N. Rd., South District, Taichung City, 40201 Taiwan; 4 Institute of Medicine, College of Medicine, Chung Shan Medical University, Taichung City, Taiwan; 5 Graduate Institute of Integrated Medicine, China Medical University, Taichung City, Taiwan

**Keywords:** Rheumatology, Risk factors

## Abstract

To study whether hydroxychloroquine (HCQ) therapy increases the risks of cataracts in patients with rheumatoid arthritis (RA). In this retrospective cohort study, 2821 treatment-naive RA patients, collected from the Longitudinal Health Insurance Database, were enrolled from 2000 to 2012 and followed up monthly until secondary cataracts were detected but no later than Dec 31, 2013. All participants were split into two groups according to the usage of HCQ in one year: the HCQ group (465 patients), with a usage duration higher than 90 days, and the non-HCQ group (465 patients), with a usage duration less than 30 days. The HCQ and non-HCQ groups were age-, sex-, complication- and drug combination-matched. There was no significant difference in survival rate between the two groups (p > 0.05). A multivariate logistic regression model was applied. Of all participants, 173 were diagnosed with secondary cataracts in both the HCQ and non-HCQ groups, with 28.8/1000 and 36.5/1000 person-years, respectively. After adjustments for other predictors, patients in the HCQ group had no increased (or decreased/equal) hazard of secondary cataract (hazard ratio (HR): 1.17; confidence interval (CI): 0.86–1.59; p > 0.05). HR analysis of HCQ usage duration, age, sex and corticosteroids showed that the CI of the adjusted HR was not statistically significant. This study showed that HCQ usage was not associated with the risk of cataracts in RA patients.

## Introduction

Rheumatoid arthritis (RA) is a common systemic inflammatory disease of small joints in which healthy cells are mistakenly attacked by the immune system, potentially causing disability^1^. Many patients suffer from RA, with a global average point and period prevalence of 0.51% and 0.56%, respectively^2^. RA is clinically diagnosed by blood and imaging tests (X-ray, CT or MRI). However, early diagnosis is difficult, and the specific causes are unknown. In addition to symptoms of joint inflammation and pain, RA can cause disease in other organs, especially the eyes, and ophthalmic involvement is usually significant^3^. The study showed that 39% of RA patients could be diagnosed with ocular diseases, such as keratoconjunctivitis sicca (KCS), uveitis, vitreitis, and cataracts^4–7^.

Cataracts are defined as opacities of the crystalline lens, which can result in vision loss and blindness^8^. Many factors contribute to the development of cataracts, such as ultraviolet exposure, advancing age, use of steroids, and underlying systemic conditions^9^. Previous studies have shown that inflammation of the eye caused by systemic risk factors, such as metabolic disorders, smoking, and autoimmune diseases, can influence the formation of cataracts^10,11^. The oxidation of lens proteins caused by inflammation of the aqueous humor is the main biochemical interpretation of this apparent association^12^. Hence, elucidating a possible relationship between rheumatoid arthritis and cataracts is of great interest.

Glucocorticoids play a physiological anti-inflammatory role in the treatment of rheumatoid arthritis, in which up to 50% of patients receive glucocorticoids in addition to disease-modifying anti-rheumatic drugs (DMARDs) and biological DMARDs. However, the long-term usage of glucocorticoids has adverse events and side effects, causing posterior subcapsular cataracts^11,13^. Hydroxychloroquine (HCQ) is one of the most widely used immunosuppressants for autoimmune diseases, with the benefits of fewer adverse effects compared to other rheumatoid drugs on the market^14^. It is also one of the recommended medicines for many national RA treatment guidelines^15,16^. HCQ is an anti-inflammatory agent that acts to prevent autoantigen presentation, decrease leukocyte activation, and reduce cytokine and prostaglandin synthesis^14^. HCQ is generally considered safe to use, and studies have reported that HCQ does not increase the risk of arrhythmias but can reduce the risk of coronary artery disease^17,18^. However, HCQ can cause eye diseases.

Early symptoms of HCQ-induced dysfunction include color vision defects, para-central visual field defects, and macular degeneration. Studies have shown that some RA patients who use HCQ also have cataracts^19,20^.

To the best of our knowledge, no studies have evaluated the association between HCQ and cataracts in patients with rheumatoid arthritis. In summary, we believe that patients with RA who use HCQ may be at risk for cataracts. To determine the effect of HCQ on cataract prevalence in RA populations, we conducted a study to investigate the association between HCQ and cataracts. The design of this study was analytical and retrospective.

## Research design and methods

### Data source

In this retrospective cohort study, the data were collected from the National Health Insurance Research Database (NHIRD), which holds all Medicare claims and medical records for 99% of the population. With this platform, clinicians are able to upload the diagnosis codes and prescriptions to the Bureau of National Health Insurance (BNHI). This study was approved by the Chung Shan Medical University Institutional Review Board (Approval number: CS19009). Furthermore, all methods were implemented in accordance with relevant guidelines and regulations.

### Study group and patient selection

**Figure 1:**
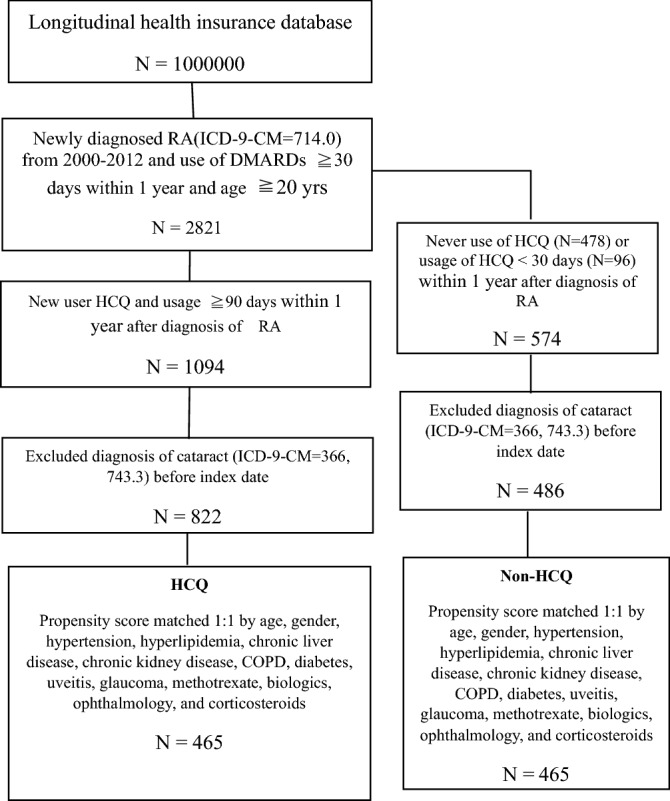
The flow chart of screening process. *HCQ *hydroxychloroquine, *DMARDs *disease modifying antirheumatic drugs, *COPD *chronic obstructive pulmonary disease.

The flowchart of data selection and classification is shown in Fig. 1. The operational definitions of the variables are detailed in Supplementary Table 1. In total, 2821 treatment-naive RA patients aged 20 years or older with ICD-9-CM = 714.0 were selected from the subdatabase of longitudinal health insurance in NHRID. One million patients were enrolled in this subdatabase from 2000 to 2012. All selected patients had taken disease modifying anti-rheumatic drugs (DMARDs) over 30 days in a year. Participants were classified into two groups according to the usage duration of HCQ: (1) the non-HCQ group, with less than 30 days of usage duration (N = 574, including the patients without hydroxychloroquine usage), and (2) the HCQ group, with more than 90 days of usage duration (N = 1094). The participants were followed up until December 31, 2013. During this time, the follow-up was stopped if secondary cataracts were detected. In this study, the index date was one year after the first diagnosis of RA, and traumatic cataracts (with ICD-9-CM = 366.2) or congenital cataracts (with ICD-9-CM = 743.3) were excluded from the study.

### Covariates and matching

The propensity score, a probability measured by logistic regression, was applied to match the characteristics of the HCQ and non-HCQ groups^21^. The propensity score of each patient was matched 1:1 according to age; sex; hypertension (ICD-9-CM = 401–405); hyperlipidemia (ICD-9-CM = 272.0–272.4); chronic liver disease (ICD-9-CM = 571); chronic kidney disease (ICD-9-CM = 585); chronic obstructive pulmonary disease (ICD-9-CM = 490–492, 494, 496); diabetes (ICD-9-CM = 250); uveitis (Supplementary Table 2); glaucoma (ICD-9-CM = 365), and usage of methotrexate, biologics, and corticosteroids. The comorbidities were diagnosed within a year after the first diagnosis of RA (one year earlier than the index date). Prescription records of the usage of methotrexate, corticosteroids, and biologics were saved during the study. Patients without referral to an ophthalmologist were also included to avoid potential screening bias.

### Statistical analysis

The HCQ and non-HCQ groups were compared using the chi-square test or independent t test. Kaplan–Meier analysis was also used to estimate the cumulative incidence of cataracts in both the HCQ and non-HCQ groups, and significance was evaluated using the log-rank test. The hazard ratio (HR) of cataracts for HCQ usage was estimated using the Cox proportional hazard model that had been adjusted for the potential confounding variables. The statistical analysis was performed using SPSS software (SPSS Inc, Chicago, IL, USA, version 18.0). Significance was defined as a p value less than 0.05.

### Ethical approval

This study was approved by the Chung Shan Medical University Institutional Review Board. Informed consent was not required if the medical data were used for research and anonymous analysis, which is in line with the Department of Health. The consent waiver statement had been approved by the Chung Shan Medical University Institutional Review Board.

## Results

### Baseline characteristics

The clinical characteristics of the participants are presented in Table 1.

**Table 1:** Demographic characteristics of hydroxychloroquine users and non-users.

	Before PSM matched		p-value	After PSM matched		p-value
	HCQ (N = 822) n (%)	Non-HCQ (N = 486) n (%)		HCQ (N = 465) n (%)	Non-HCQ (N = 465) n (%)	
Age, years			0.030			0.393
20–65	735 (89.4)	452 (93)		425 (91.4)	432 (92.9)	
≥ 65	87 (10.6)	34 (7)		40 (8.6)	33 (7.1)	
Mean ± SD	49.7 ± 13	47.3 ± 11.9	0.001*	48.2 ± 12.4	47.9 ± 11.6	0.682
Gender			< 0.001*			0.682
Female	616 (74.9)	301 (61.9)		294 (63.2)	300 (64.5)	
Male	206 (25.1)	185 (38.1)		171 (36.8)	165 (35.5)	
Hypertension	148 (18)	75 (15.4)	0.232	79 (17)	75 (16.1)	0.724
Hyperlipidemia	54 (6.6)	37 (7.6)	0.473	38 (8.2)	35 (7.5)	0.715
Chronic liver disease	66 (8)	32 (6.6)	0.338	37 (8)	32 (6.9)	0.532
Chronic kidney disease	12 (1.5)	6 (1.2)	0.735	4 (0.9)	6 (1.3)	0.525
COPD	35 (4.3)	15 (3.1)	0.286	12 (2.6)	13 (2.8)	0.839
Diabetes	52 (6.3)	27 (5.6)	0.572	32 (6.9)	26 (5.6)	0.416
Uveitis^†^	5 (0.6)	5 (1)	0.514	4 (0.9)	5 (1.1)	1
Glaucoma^†^	9 (1.1)	3 (0.6)	0.551	1 (0.2)	3 (0.6)	0.624
Methotrexate	406 (49.4)	230 (47.3)	0.470	227 (48.8)	224 (48.2)	0.844
Biologics	97 (11.8)	34 (7)	0.005*	38 (8.2)	34 (7.3)	0.624
Ophthalmology	513 (62.4)	292 (60.1)	0.403	284 (61.1)	280 (60.2)	0.788
Corticosteroids	678 (82.5)	400 (82.3)	0.935	383 (82.4)	383 (82.4)	1

^†^Fisher's exact test.

*p < 0.01.

The mean time of follow-up was 5.5 ± 3.7 years in the hydroxychloroquine group and 6.0 ± 3.8 years in the non-hydroxychloroquine group (p > 0.05).The heterogeneity between the HCQ and non-HCQ groups was balanced by matching the propensity score. With the propensity score matching, there was no statistical significance for age, sex, or the number of participants with biological therapy. In the balanced dataset, 63.9% (n = 594) of patients were female, and 92.2% (n = 857) of patients were 20–65 years old.

### Primary endpoint

Overall, 173 participants were diagnosed with secondary cataracts, including 80 in the non-HCQ group and 93 in the HCQ group. The incidence of cataracts was estimated using the Cox proportional hazards model (Table 2). By recording the number of endpoints, we estimated the incidence of events per 1000 person-years (incidence density). The crude HR showed that the comorbidities of hypertension, hyperlipidemia, chronic kidney disease, COPD and diabetes also increased the risks of cataracts, and the HR of cataracts was 1.28 (95% CI, 0.95–1.73) for HCQ usage. After the data correction of age, sex, hypertension, hyperlipidemia, chronic liver disease, chronic kidney disease, COPD, diabetes, uveitis, methotrexate, corticosteroids, biological agents and ophthalmology, only age, COPD, diabetes, and biologics use showed significantly different HR (95% CI) values. The adjusted HRs (95% CI) of male patients, COPD patients, and diabetes patients were 3.15 (2.07–4.80), 2.02 (1.05–3.91), and 2.59 (1.58–4.24), respectively. However, the adjusted HR (95% CI) of biological medicine usage was 0.33 (0.13–0.82). The adjusted HR of cataracts between the HCQ and non-HCQ groups was 1.17 (95% CI, 0.86–1.59, P > 0.05). The statistical analysis demonstrated in Table 2 shows that HCQ usage did not increase the risk of cataracts in RA patients.

**Table 2:** Cox proportional hazard model for risk of cataract.

	No. of cataract event	Observed person-years	Incidence density (per 1000 person-years)	Crude HR (95% C.I.)	Adjusted HR^†^ (95% C.I.)
Hydroxychloroquine					
No	80	2775	28.8	1	1
Yes	93	2551	36.5	1.28 (0.95–1.73)	1.17 (0.86–1.59)
Age					
20–65	140	5018	27.9	1	1
≥65	33	309	106.9	**3.89 (2.65–5.69)**	**3.15 (2.07–4.80)**
Gender					
Female	104	3439	30.2	1	1
Male	69	1888	36.5	1.21 (0.89–1.64)	1.21 (0.89–1.65)
Hypertension	43	763	56.4	**1.98 (1.40–2.79)**	1.29 (0.86–1.93)
Hyperlipidemia	19	331	57.4	**1.86 (1.16–3.01)**	1.28 (0.76–2.14)
Chronic liver disease	13	416	31.3	0.96 (0.55–1.69)	0.99 (0.55–1.77)
Chronic kidney disease	2	38	52.7	1.63 (0.40–6.57)	0.82 (0.20–3.45)
COPD	10	159	62.8	**1.99 (1.05–3.77)**	**2.02 (1.05–3.91)**
Diabetes	24	243	98.6	**3.38 (2.19–5.22)**	**2.59 (1.58–4.24)**
Uveitis	3	52	57.2	1.78 (0.57–5.58)	2.68 (0.84–8.56)
Methotrexate	76	2633	28.9	0.80 (0.60–1.09)	1.00 (0.73–1.37)
Biologics	5	445	11.2	**0.33 (0.14–0.81)**	**0.33 (0.13–0.82)**
Ophthalmology	108	3748	28.8	**0.69 (0.51–0.95)**	0.89 (0.64–1.24)
Corticosteroids	141	4738	29.8	**0.54 (0.37–0.80)**	0.52 (0.34–0.78)

Bold font represents statistical significance (p < 0.05).

^†^Adjusted for age, gender, hypertension, hyperlipidemia, chronic liver disease, chronic kidney disease, COPD, diabetes, uveitis, methotrexate, biologics, ophthalmology, and corticosteroids.

**Figure 2:**
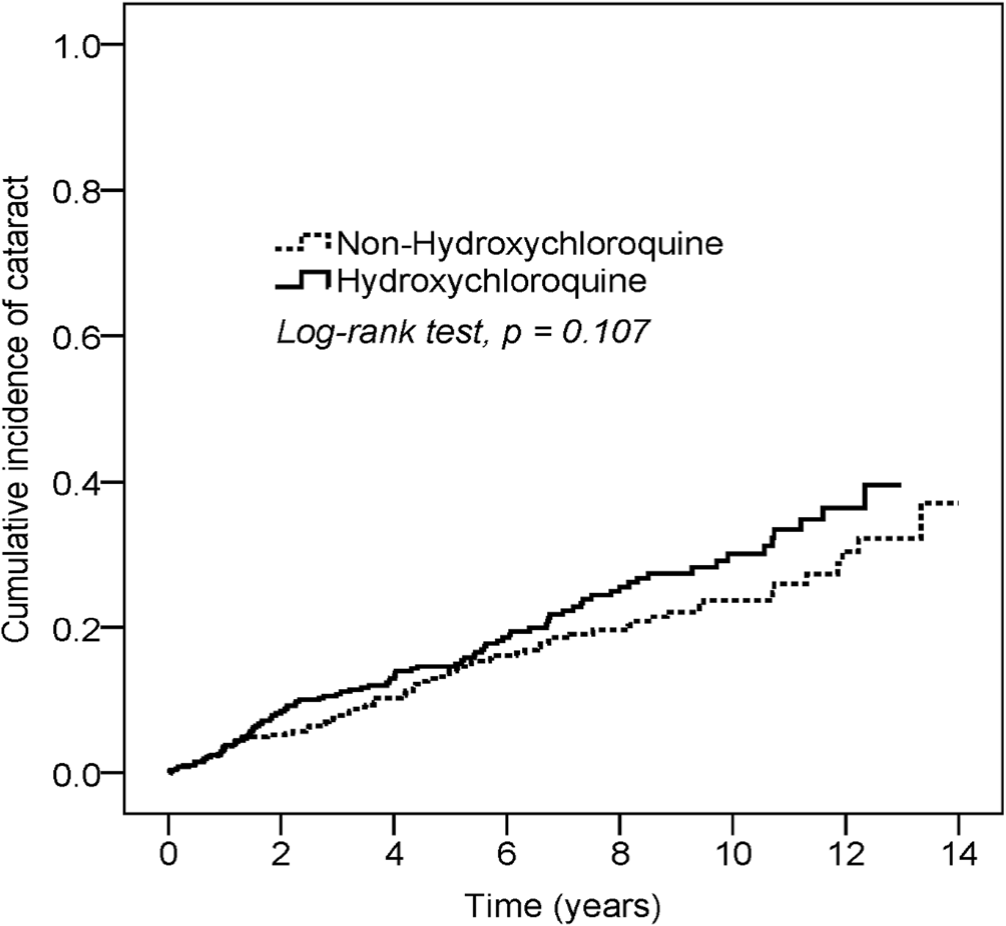
Cumulative incidence rate of cataract for hydroxychloroquine users and non-users in RA patients.

Figure 2 reveals the cumulative incidence rates of cataracts for hydroxychloroquine users and nonusers. The log-rank test showed no significant differences in the survival rate between the two groups (P > 0.05), indicating no significant differences in the cumulative incidence rate between the two groups.

### HCQ dose effect

Table 3 shows the results of the Cox proportional hazard model analysis of the effects of HCQ usage duration. The incidence of cataracts per 1000 person-years (incidence density) was also estimated in the subgroups. Compared to the non-HCQ group, the HCQ usage duration was not different, no matter whether it was 90–180 days, 180–270 days, or more than 270 days. And the CI of the adjusted HR was still not statistically significant. Based on the above analysis, we concluded that the HCQ usage duration did not impact the risk of cataracts.

**Table 3:** Cox proportional hazard model among different Hydroxychloroquine usage duration.

	No. of cataract event	Observed person-years	Incidence density (per 1000 person-years)	Crude HR (95% C.I.)	Adjusted HR^†^ (95% C.I.)
Hydroxychloroquine					
No	80	2775	28.8	1	1
90–180 days	37	914	40.5	1.41 (0.95–2.08)	1.19 (0.79–1.78)
180–270 days	23	732	31.4	1.11 (0.69–1.76)	1.02 (0.64–1.64)
≥ 270 days	33	905	36.5	1.29 (0.86–1.93)	1.29 (0.85–1.97)

^†^Adjusted for age, gender, hypertension, hyperlipidemia, chronic liver disease, chronic kidney disease, COPD, diabetes, uveitis, methotrexate, biologics, ophthalmology, and corticosteroids.

### Subgroup analyses

Table 4 shows the subgroup analysis comparing the differences in age, sex and corticosteroids between the two groups. The Cox proportional hazard model was used for the analysis. HR was calculated for RA patients by age, sex,and corticosteroids. The adjusted HR (95% CI) between HCQ users and nonusers was1.35 (0.96–1.90) for those aged 20–65 years. For patients with RA aged 65 years or older, the adjusted HR (95% CI) during follow-up was 0.71 (0.31–1.63). The adjusted HR (95% CI) was 0.87(0.53–1.44) for males and 1.44 (0.97–2.15) for females. The adjusted HR (95% CI) was 1.2(0.85–1.68) for use with corticosteroids and 1.32 (0.62–2.79) for use without corticosteroids. Therefore, there were no significant differences between the subgroups. We concluded that there were no significant changes in the risk of cataracts between groups of rheumatoid arthritis patients based on age sex or corticosteroids.

**Table 4:** Subgroup analysis of Cox proportional hazard model.

	Hydroxychloroquine		Non-Hydroxychloroquine		HR (95% C.I.)
	N	No. of Cataract	N	No. of Cataract	
Age^†^					
20–65	425	76	432	64	1.35 (0.96–1.90)
≥ 65	40	17	33	16	0.71 (0.31–1.63)
Gender^‡^					
Female	294	60	300	44	1.44 (0.97–2.15)
Male	171	33	165	36	0.87 (0.53–1.44)
Corticosteroids^¶^					
No	82	17	82	15	1.32 (0.62–2.79)
Yes	383	76	383	65	1.20 (0.85–1.68)

^†^Adjusted for age, gender, hypertension, hyperlipidemia, chronic liver disease, chronic kidney disease, COPD, Diabetes, methotrexate, biologics, ophthalmology, and corticosteroids.

^‡^Adjusted for age, gender, hypertension, hyperlipidemia, chronic liver disease, COPD, Diabetes, methotrexate, biologics, ophthalmology, and corticosteroids.

^¶^Adjusted for age, gender, hypertension, hyperlipidemia, chronic liver disease, diabetes, methotrexate, and ophthalmology.

## Discussion and conclusion

To the best of our knowledge, this study is the first to investigate the correlation between the risks of cataracts and HCQ therapy. The statistical analysis showed that HCQ therapy did not increase the risk of cataracts in RA patients. The RA patients used in this study were selected from the NHIRD, which records the clinical information of approximately 99% of Taiwanese residents. With adjustments to remove confounders, the statistical analysis supported the hypothesis that there was no relationship between HCQ therapy and the risks of cataracts.

RA is a common autoimmune inflammatory disease that can affect other organs throughout the body. Recent studies have found that RA can also increase the incidence of endometriosis^22^. According to Wei et al., HPV infection may be associated with a higher risk of RA progression^9^. The immunomodulatory processes of some systemic diseases may mimic the pathological changes that characterize the eyes in RA. These include collagenases, such as matrix metalloproteinase, neutrophil- and macrophage-mediated attacks, the deposition of immune complexes, various components of adaptive immunity and cytokines, and the complement cascade^23^. In addition, several systemic inflammatory mediators have been demonstrated to be relevant. A previous study showed that age-related cataracts were related to TNFα, IL-6, intracellular adhesion molecule-1, and high-sensitivity CRP^24^. In our study, we focused on age, sex, comorbidities and concomitant medications. After data adjustments, the HR (95% CI) of age, COPD, diabetes,and biological medicine usage was significantly different. The use of HCQ was excluded. In addition, comparing the differences in HCQ usage duration, age, sex and corticosteroids between the two groups in the subgroup analysis, here was no significant difference compared with the control group.

Cataracts are also associated with certain medications, such as glucocorticoids. A meta-analysis and systematic review of the association between cataract deterioration and corticosteroids use is supported by current data, but the risk cannot be quantified in patients with RA^25^. Cataracts are known sequelae of juvenile RA-related uveitis and are believed to be due to corticosteroid use and chronic inflammation^26^. Studies have shown that cataracts in SLE patients can be caused by many factors, including glucocorticoids, systolic blood pressure and disease activity^11^. Lee HJ and Kim SJ reported that sudden vision loss could be caused by the disease itself and medicine in systemic lupus erythematosus patients^27^. Our study had included the factor of corticosteroids, but the conclusion was that the HCQ usage does not increase the risk of cataract, whether combined or not, and also showed that biologic therapy could reduce the risks of secondary cataracts in elderly patients (older than 65 years old) with chronic obstructive pulmonary disease (COPD) or diabetes.

According to a prospective and cross-sectional survey, 4 out of 60 RA patients who had received HCQ therapy over 6 months were diagnosed with secondary cataracts 6 months later^19^. However, the major limitation of this study was that the number of samples was small. In addition, the conclusion of this study suggested that dosing regimen and the total dose of HCQ were related to the risk of retinopathy, and there was no evidence that HCQ caused cataract in the research. In another retrospective study, 2867 rheumatic patients taking HCQ were reviewed, but only 31 patients were diagnosed with blindness or macular degeneration, and only 9% had cataracts. Seventeen of the 31 patients had visual impairment. Each patient was considered to originate from multiple factors, and no one was considered to be directly related to HCQ toxicity^20^. This study mainly focused on blindness in rheumatic patients but did not further investigate the correlation between cataracts and HCQ usage. In our study, the endpoint was finding cataracts, and the statistical analysis showed that there was no significant difference between the HCQ and non-HCQ groups (36.2 vs. 28.8 per 1000 person-years; adjusted HR = 1.20, 95% CI 0.88 to 1.63). We found that HCQ usage did not increase the risk of cataracts in RA patients.

HCQ usage, which is an important immunosuppressant, should not be replaced by considering only the risk of cataracts but should ignore the relevance to age, sex, and HCQ usage duration. The results of this study are essential for physicians to use HCQ. However, for complex patients with multiple complications and severe RA patients with high inflammatory markers, a comprehensive evaluation of the condition and a referral to ophthalmologists are still highly recommended before taking HCQ. More longitudinal studies on patients with other diseases and taking HCQ are necessary. In addition, a combination of multiple medicines, including NSAIDs, DMARDs, glucocorticoids and biological medicines, is commonly used to treat RA in the clinic. Further studies are also necessary to determine whether other medicines increase the risks of cataracts in RA patients.

There were several limitations in our study. First, information on disease activity was lacking. Second, clinical activity indices (such as radiation data, erythrocyte sedimentation rate, rheumatoid factor, and C-reactive protein) may also be related to cataracts, but this was beyond the scope of our study. Moreover, some confounders were not recorded in the NHIRD, such as body weight and lifestyle habits (e.g., smoking and serum lipids). Finally, subclinical patients who did not seek medical attention were not included. We included patients who were present for eye disease during the study period to reduce potential screening bias.

To date, many studies have discussed ophthalmic symptoms of rheumatic patients during the use of HCQ, such as blindness and cataract maculopathy^27,28^. Compared with SLE, there has been relatively little research on this subject in RA. Comprehensive studies with longitudinal follow-ups are needed to confirm any possible association between hydroxychloroquine and the risk of cataracts in RA patients. Although this research suggested that HCQ did not increase the incidence of cataracts in RA patients, it may depend on the dose and time of treatment involved and other risk factors. However, in clinical work, rheumatologists still need to cooperate with ophthalmologists and screen early signs of eye disease, including cataracts.

## Supplementary Information

Supplementary Table 1.

Supplementary Table 2.

## Data Availability

The datasets used and/or analysed during the current study are available from the corresponding author on reasonable request.

## Abbreviations

HCQ: Hydroxychloroquine
RA: Rheumatoid arthritis
NHIRD: National Health Insurance Research Database
BNHI: Bureau of National Health Insurance
NSAIDs: Non-steroidal anti-inflammatory drugs
DMARDs: Disease-modifying anti-rheumatic drugs
