# Supplementary material for: Hydroxychloroquine is neutral on incidental cataracts in patients with rheumatoid arthritis
Source: Sci Rep. 2023 Apr 5;13:5576. doi: 10.1038/s41598-023-32297-x (PMC10076357; doi:10.1038/s41598-023-32297-x)
Supplement: Supplementary file 1 — Supplementary Table 1. [file 41598_2023_32297_MOESM1_ESM.docx]

| **The variable name** | Investigate the operational definition |
| --- | --- |
| **Dependent variable** |  |
| Cataract | ICD-9-CM codes (ICD=366, except 366.2 [traumatic cataract] and 743.3 [congenital cataract]) by at least one time hospitalization with a cataract diagnosis or a one-time outpatient diagnosis by an ophthalmologist. |
| Index date | Case use Hydroxychloroquine date/comparison:RA date |
| End date | 1.Hypertension date 2.2013/12/31 3.Drop out of Longitudinal Health Insurance Database |
| **Independent variable** |  |
| Period of Study | 2000/1/1~2013/12/31(annual data) |
| Rheumatoid Arthritis | 2000-2010 Once a diagnosis of Rheumatoid Arthritis(ICD-9-CM=714.0) |
| Hydroxychloroquine | Hydroxychloroquine was used for at least ≧90 days during screening |
| Gender | 0 female 1 male |
| Age | Index date age |
| Hypertension | Observation starting point (including) medical treatment in the previous year meets the definition(outpatient 2/hospitalization 1)  ICD-9-CM=401-405 0 no 1 yes |
| Hyperlipidemia | Observation starting point (including) medical treatment in the previous year meets the definition(outpatient 2/hospitalization 1)  ICD-9-CM=272.0-272.4 0 no 1 yes |
| Chronic liver disease | Observation starting point (including) medical treatment in the previous year meets the definition(outpatient 2/hospitalization 1)  ICD-9-CM=571 0 no 1 yes |
| Chronic kidney disease | Observation starting point (including) medical treatment in the previous year meets the definition(outpatient 2/hospitalization 1)  ICD-9-CM=585 0 no 1 yes |
| COPD | Observation starting point (including) medical treatment in the previous year meets the definition(outpatient 2/hospitalization 1)  ICD-9-CM=490-492, 494, 496 0 no 1 yes |
| DM | Observation starting point (including) medical treatment in the previous year meets the definition(outpatient 2/hospitalization 1)  ICD-9-CM=250 0 no 1 yes |
| Uveitis | Observation starting point (including) medical treatment in the previous year meets the definition(outpatient 2/hospitalization 1)  ICD-9-CMSupplementary Tabel 2) |
| Glaucoma | Observation starting point (including) medical treatment in the previous year meets the definition(outpatient 2/hospitalization 1)  ICD-9-CM=365 0 no 1 yes |
| Methotrexate (MTX) | Whether or not it was used during the study observation period Methotrexate |
| Biologics | Whether or not it was used during the study observation period Etanercept, adalimumab, golimumab, rituximab |
| Ophthalmology | Screening for visits to an ophthalmologist during the observation period |
| Corticosteroids | Whether or not it was used during the study observation period , tablets or injections. |

Supplementary Tabel 1 Investigate the operational definition of variables **name**
