# Supplementary material for: Hydroxychloroquine is neutral on incidental cataracts in patients with rheumatoid arthritis
Source: Sci Rep. 2023 Apr 5;13:5576. doi: 10.1038/s41598-023-32297-x (PMC10076357; doi:10.1038/s41598-023-32297-x)
Supplement: Supplementary file 2 — Supplementary Table 2. [file 41598_2023_32297_MOESM2_ESM.docx]

Supplementary Tabel 2 ICD-CM Codes 2001 version for uveitis

| **Anterior uveitis** | | **Intermediate uveitis** | |
| --- | --- | --- | --- |
| Herpes Zoster iridbcyclitis | 053.22 | Pars planitis | 363.21 |
| Herpes simplex iridocyclitis | 054.44 | Vitreous abscess | 360.04 |
| Syphilitic iridbcyclitis (secondary) | 091.52 |  |  |
| Acute and subacute iridocyclitis | 364.0 | **Posterior uveitis** |  |
| Acute and subacute iridocyclitis, unspecified | 364.00 | Syphilitic chorioretinitis (secondary) | 091.51 |
| Primary iridocyclitis | 364.01 | Syphilitic disseminated retinochoroiditis | 094.83 |
| Recurrent iridocyclitis | 364.02 | Retinitis, infection by histoplasma capsulatum | 115.02 |
| Secondary iridocyclitis, infectious | 364.03 | Retinitis, infection by histoplasma duboisii | 115.12 |
| Secondary iridocyclitis, noninfectious | 364.04 | Retinitis. infection by histoplasmosis, unspecified | 115.92 |
| Hypopyon | 364.05 | Chorioretinitis due to toxoplasmosis | 130.2 |
| Chronic iridocyclitis | 364.1 | Retinal vasculitis | 362.18 |
| Chronic iridocyclitis, unspecified | 364.10 | Focal chorioretinitis and focal retinochoroiditis | 363.0 |
| Chronic iridocyclitis in disease classified elsewhere | 364.11 | Focal chorioretinitis,unspecified | 363.00 |
| Certain types ofiridocyclitis | 364.2 | Focal choroiditis and chorioretinitis,juxtapapillary | 363.01 |
| Fuch's heterchromic cyclitis | 364.21 | Focal choroiditis and chorioretinitis of other posterior pole | 363.03 |
| Glaucomatocyclitic crises | 364.22 | Focal choroiditis and chorioretinitis,peripheral | 363.04 |
| Lens-induced iridocyclitis | 364.23 | Focal retinitis and retinochoroiditis,juxtapapillary | 363.05 |
| Unspecified iridocyclitis | 364.3 | Focal retinitis and retinochoroiditis, macular | 363.06 |
|  |  | Focal retinitis and retinochoroiditis of other posterior pole | 363.07 |
| **Panuuveitis** |  | Focal retinitis and retinochoroiditis, peripheral | 363.08 |
| Purulent endophthalmitis | 360.0 | Disseminated chorioretinitis and retinochoroiditis | 363.1 |
| Purulant endophthalmitis unspeccified | 360.00 | Disseminated chorioretinitis,unspecified | 363.10 |
| Acute endophthalmitis | 360.01 | Disseminated choroiditis and chorioretinitis, posterior pol | 363.11 |
| Panophthalmitis | 360.02 | Disseminated choroiditis and chorioretinitis, peripheral | 363.12 |
| Chronic endophthalmitis | 360.03 | Disseminated choroiditis and chorioretinitis, generalized | 363.13 |
| Other endophthalmitis | 360.1 | Disseminated retinitis and retinochoroiditis, metastatic | 363.14 |
| Sympathetic uveitis | 360.11 | Disseminated retinochoroiditis, pigment epitheliopathy | 363.15 |
| Panuveitis | 360.12 | Other forms of chorioretinitis and retinochoroiditis | 363.2 |
| Parasitic endophthalmitis Nos | 360.13 | Chorioretinitis, unspecified | 363.20 |
| Ophthalmia nodosa | 360.14 | Harada's disease | 363.22 |
| Other endophthalmitis | 360.19 | *Vogt-Koy*anagi syndrome | 364.24 |
| Uveitis due to secondary syphilis | 091.5 |  |  |
| Syphilitic uveitis, unspecified | 091.50 |  |  |
